# Combined analgesics in (headache) pain therapy: shotgun approach or precise multi-target therapeutics?

**DOI:** 10.1186/1471-2377-11-43

**Published:** 2011-03-31

**Authors:** Andreas Straube, Bernhard Aicher, Bernd L Fiebich, Gunther Haag

**Affiliations:** 1 Department of Neurology, Klinikum Großhadern, Ludwig-Maximilians-University, D-81377 Munich, Germany; 2 Boehringer Ingelheim Pharma GmbH&Co. KG, Binger-Str. 173, D-55216 Ingelheim am Rhein, Germany; 3 Dept. of Psychiatry and Psychotherapy, Universitätsklinikum Freiburg, Hauptstr. 5, D-79104 Freiburg, Germany; 4 Michael-Balint Klinik, Hermann-Voland Str. 10, D-78126 Königsfeld im Schwarzwald, Germany

**Keywords:** analgesics, fixed-dose combinations, headache, multi-target therapeutics, migraine, over-the-counter (OTC), pain, side effects, tension-type headache

## Abstract

**Background:**

Pain in general and headache in particular are characterized by a change in activity in brain areas involved in pain processing. The therapeutic challenge is to identify drugs with molecular targets that restore the healthy state, resulting in meaningful pain relief or even freedom from pain. Different aspects of pain perception, i.e. sensory and affective components, also explain why there is not just one single target structure for therapeutic approaches to pain. A network of brain areas ("pain matrix") are involved in pain perception and pain control. This diversification of the pain system explains why a wide range of molecularly different substances can be used in the treatment of different pain states and why in recent years more and more studies have described a superior efficacy of a precise multi-target combination therapy compared to therapy with monotherapeutics.

**Discussion:**

In this article, we discuss the available literature on the effects of several fixed-dose combinations in the treatment of headaches and discuss the evidence in support of the role of combination therapy in the pharmacotherapy of pain, particularly of headaches. The scientific rationale behind multi-target combinations is the therapeutic benefit that could not be achieved by the individual constituents and that the single substances of the combinations act together additively or even multiplicatively and cooperate to achieve a completeness of the desired therapeutic effect.

As an example the fixesd-dose combination of acetylsalicylic acid (ASA), paracetamol (acetaminophen) and caffeine is reviewed in detail. The major advantage of using such a fixed combination is that the active ingredients act on different but distinct molecular targets and thus are able to act on more signalling cascades involved in pain than most single analgesics without adding more side effects to the therapy.

**Summary:**

Multitarget therapeutics like combined analgesics broaden the array of therapeutic options, enable the completeness of the therapeutic effect, and allow doctors (and, in self-medication with OTC medications, the patients themselves) to customize treatment to the patient's specific needs. There is substantial clinical evidence that such a multi-component therapy is more effective than mono-component therapies.

## 1. Background

Almost everybody will probably suffer from acute pain during their lifetime. Pain is a fundamental and central life experience, a counterbalance to pleasure, a warning of danger, and a reminder to protect injured limbs and tissues while they heal [1]. The perception of pain is essential for survival and thus it is not surprising that humans with loss of pain sensation due to a mutation within the Na 1.7 channel gene [2] die at a young age. While in the past pain was seen as a relatively simple symptom, it is becoming increasingly clear that, from the molecular-biological mechanisms to the impact on social systems, it is, in fact, a highly complex phenomenon [3]. The sensation of pain has several dimensions: in addition to sensory perception, there is always also an emotional aspect and a spiritual aspect of pain.

These different aspects of pain perception also explain why there is not just one single target structure for therapeutic approaches to pain. A network of brain areas is involved in pain perception and pain control. In addition to the ascending pain pathways, which can be differentiated into a lateral pathway, more responsible for the spatial localisation of pain, and a medial, limbic pathway, more engaged in the affective rating of pain (e.g. pain during delivery compared to pain due to social misconduct) [4], a descending pain control system also exists. This descending antinociceptive system is responsible for the often unconscious control of nociceptive inflow. This antinociceptive system includes areas in the anterior cingulated cortex, hypothalamus, periaquaductal grey area, and rostral medullar area as well as descending pathways to the dorsal spinal horn. Activity in these areas can be connected to the effects seen in placebo and nocebo responses [5].

This diversification of the pain system also explains why a wide range of different molecular substances can be used in the treatment of different pain states. Specific drugs directed at individual molecular targets are often found to be less effective at treating disease or disease symptoms than multi-target therapeutics. This particularly applies in the case of pain therapy. It would appear that the tendency, which was prevalent in the past, to view cellular causation as conforming to simple linear patterns in which macro-scale effects are specified by micro-scale structures [6] needs to be revised. The complexity of the molecular-biological mechanisms requires precise (multi)target modulation [7]. The limitations of many monotherapies can be overcome by attacking the disease system via multiple pathways [8]. In many important therapeutic areas such as diabetes, infectious disease, asthma, hypertension, depression, anxiety disorder, cancer pain therapy, and, as discussed here, pain therapy, multi-component drugs are now standard [9]. In this article, we describe the scientific evidence for the superior efficacy of fixed-dose combinations [10] and discuss their role in the pharmacotherapy of pain and particularly of headaches.

## 2. Discussion

### 2.1 Multi-target approach

It is important to note that the distinction between a single drug with a single pharmacological activity and a combination drug with combined pharmacological activities is not absolute [11]. It is known from various analgesics and NSAIDs (non steroidal anti inflammatory drugs) that different mechanisms of their analgesic effect are discussed in the most diverse of pain models (e.g. molar extraction, postoperative postpartum pain, back pain, migraine, and tension-type headache) and in clinical pain states. This is the case, for instance, for paracetamol [12], acetylsalicylic acid (ASA) [13] and ibuprofen [14], for which it is assumed, like other NSAIDs, that they act at both peripheral and spinal/higher nervous centres and that these involve prostaglandin-independent as well as prostaglandin-dependent mechanisms [14]. Moreover, ibuprofen assumes an intermediate position between a single drug with a single pharmacological activity and a combination drug insofar as a racemate is concerned, i.e. a "combination" of the (S)-(+) enantiomer and the (R)-(-) enantiomer. In this regard, the (S)-(+) enantiomer is appreciably more potent as an inhibitor of prostaglandin production than the (R)-(-) form [14].

However, in general a combination therapy or a multi-target approach is understood as a combination of two or more active pharmaceutical ingredients [10] with complementary mechanisms of action, which extends the range of therapeutic options in the treatment of almost every human disease [11].

### 2.2 Multi-target therapeutics with two or more drugs

#### 2.2.1 Indomethacin + prochlorperazine + caffeine

The fixed combination of indomethacin (a potent, non-selective inhibitor of cyclooxygenase enzymes), prochlorperazine (a phenothiazine antiemetic with analgesic properties), and caffeine (a methylxanthine, and an effective analgesic adjuvant), has been a frequently used medication for the acute treatment of migraine and tension-type headache (TTH) in Italy for over 30 years [15]. In experimental animal models, this combination had an antihyperalgesic activity that was significantly superior to that of its single components [16]. The fixed combination was able to abolish the peripheral sensitization induced by kainic acid and the central sensitization induced by N-methyl-D-aspartate (NMDA) in in vivo models of hyperalgesia, while sumatriptan was not able to reverse either the kainic acid-induced or the NMDA-induced hyperalgesia [17].

In a mice model of the abdominal constriction test, the fixed combination and sumatriptan at analgesic doses exerted their central antinociceptive action independently of the Gi proteins, and the efficacy of the combination was statistically superior to that of sumatriptan [18]. In a double-blind, double-dummy, randomised, parallel group, multicenter study, the fixed combination and sumatriptan (50 mg) did not significantly differ in the acute treatment of migraine attacks in terms of the primary efficacy endpoint [15]. In a similar study on the treatment of episodic tension-type headache, the fixed combination was significantly superior to the control substance nimesulide (a sulfonamide compound with antiinflammatory, analgesic and antipyretic effects) at 100 mg in the second headache episode, but not in the first episode examined [19].

#### 2.2.2 Triptans + NSAIDs or other drugs

Although the introduction of triptans in the 1980s opened up a new therapeutic option in particular for migraine, in a publication entitled "Beyond monotherapy: rational polytherapy in migraine" in 1998, Peroutka [20] pointed out that, although monotherapeutic approaches are effective in many migraineurs, they do not provide rapid, consistent and complete relief in all of them. Using the pharmaceutical prescriptions database of two consecutive years in a regional Health Authority in Italy, a low percentage of triptan users and a low rate of utilization, associated with a high percentage of discontinuation and new utilization, were observed, without any substantial increase in triptan utilization over the years. All of these data probably do not support optimal satisfaction with triptan therapy [21]. This confirms data from the southern district of "Clalit health services," a large Israeli HMO (Health Maintenance Organization), showing that many migraine patients choose not to use triptans after their first experience with the drug [22]. Therefore, if monotherapy is suboptimal, it logically follows that concurrent therapy (i.e., polytherapy) aimed at two or three biological systems should be more efficacious than a therapy modulating only a single system [20]. Krymchantowski agrees with this and, on the basis of a whole series of clinical studies (Table 1), calls for "breaking the paradigm of monotherapy" [23].

**Table 1:** Clinical studies of triptan + NSAID combinations in the treatment of migraine (published 1999-2009)

Authors/year of publication	Study type	Type of combination (dosage)	Dosage regime
Krymchantowski et al. 1999 [24]	retrospective clinical case series	sumatriptan (100 mg) + tolfenamic acid 200 mg	single dose/attack (number of treated attacks per patient not reported)

Krymchantowski 2000 [25]	open-label study	sumatriptan (100 mg) + naproxen sodium (550 mg)	single dose (3 attacks treated)

Smith et al. 2005 [26]	multicenter, randomized, double-blind, double-dummy, placebo-controlled, four-arm study	sumatriptan (50 mg) + naproxen sodium (500 mg) sumatriptan (50 mg) naproxen sodium (500 mg) placebo	single dose

Brandes et al. 2007 [27]	two replicate, randomized, double-blind, single attack, parallel-group studies	sumatriptan (85 mg) + naproxen sodium (500 mg) sumatriptan (85 mg) naproxen sodium (500 mg) placebo	single dose

Lipton et al. 2009 [28]	two identical randomized, placebo-controlled, crossover studies	sumatriptan (85 mg) + naproxen sodium (500 mg) placebo	single dose (4 attacks treated) report of 2 clinical studies

Krymchantowski & Barbosa 2002 [29]	open label pilot study	rizatriptan (10 mg) + rofecoxib (25 mg) rizatriptan (10 mg)	single dose (3 attacks treated)

Krymchantowski & Bigal 2004 [30]	randomized, open-label, cross-over study	rizatriptan (10 mg) + tolfenamic acid (200 mg) rizatriptan (10 mg) + rofecoxib (50 mg) rizatriptan (10 mg)	single dose (2 attacks treated)

Krymchantowski et al. 2006 [31]	double-blind, randomized, cross-over study	rizatriptan (10 mg) + trimebutine (200 mg) rizatriptan (10 mg)	single dose (2 attacks treated)

Freitag et al. 2008 [32]	randomized, double-blind, placebo-controlled study	rizatriptan (10 mg) + acetaminophen (1000 mg) rizatriptan (10 mg) acetaminophen (1000 mg) placebo	single dose

Schoenen et al. 2008 [33]	multicenter, double-blind, double-dummy, cross-over pilot study	almotriptan (12.5 mg) + aceclofenac (100 mg) almotriptan (12.5 mg) + placebo	single dose

From a retrospective clinical case series, the observation was made that in migraine patients for whom headache recurrence within 24 h frequently occurred following attack treatment with sumatriptan, the combination of sumatriptan with an NSAID (tolfenamic acid 200 mg) led to a reduction in the recurrence rate [24]. This reduction in the recurrence rate was also shown for the combination of sumatriptan with naproxen sodium in an open-label study [25].

These observations were confirmed in randomized controlled clinical trials, which demonstrated that multimechanism acute therapy for migraine, combining a triptan (sumatriptan) and an analgesic (naproxen sodium) offers improved clinical benefits over monotherapy with these selected standard antimigraine treatments [26]. The benefit of this combination existed not only in the lowest headache recurrence rate but also in significantly superior pain relief (2-hour pain response) [26]. In two further clinical studies, the fixed combination of sumatriptan (85 mg) plus naproxen sodium (500 mg) as a single tablet resulted in more favourable clinical benefits compared with either monotherapy [27]. The consistency of these benefits over multiple migraine attacks was recently shown in two further clinical studies [28].

Such a benefit was also shown in an open-label study on the combination of rizatriptan with the COX-2 inhibitor rofecoxib. The combination reduced recurrence rates compared to rizatriptan monotherapy, [29]. A similar result was shown for the combination of rizatriptan and tolfenamic acid, a traditional NSAID [30]. In addition, acetaminophen has been assessed as part of combination therapy with rizatriptan [32]. The combination proved superior to both acetaminophen and placebo, but failed to achieve superiority over rizatriptan alone, which the authors attributed to the low number of cases and consequently insufficient power [32]. Therefore, Krymchantowski and Bigal conclude that recent evidence that has been gathered on combination therapies utilizing rizatriptan plus NSAID and even gastrokinetic drugs, such as trimebutine, point to a future of more effective combined therapy for migraine [34]. Targeting other associated mechanisms, such as inflammation and gastric stasis, that occur during the migraine attack, by combining triptans with anti-inflammatory and/or prokinetic agents may possibly enhance the clinical outcomes [35].

Even though the clinical data are still limited at present, it can nevertheless be stated that a multi-target combination therapy with a triptan plus an NSAID is more effective in acute migraine treatment than monotherapy with either drug alone. This superior effectiveness relates not only to a more successful avoidance of headache recurrence, but equally to superior pain relief (2-hour pain response). These observations are in good agreement with the basic scientific results showing that in the course of a migraine attack not only triptan responsible processes like activation of 5-HT 1D receptors take place but also an up-regulation of cyclooxgenase 2 can be detected [36].

### 2.3 Caffeine in multi-target pain therapeutics

The use of multi-target pain therapeutics in the treatment of migraine is not new. Ergotamine tartrate has typically been prescribed as a combination agent with caffeine and other adjunctive therapeutic agents [32]. Caffeine has been used in combination with mild analgesics for many decades, with its utility deriving from its adjuvant properties. Sawynok and Yaksh [37] presented a review of data from 27 clinical studies in which caffeine was examined in combination with antipyretic analgesics and found that there are only a few investigations that have defined caffeine actions as an analgesic when given alone [37]. In a study by Camann et al. [38], oral caffeine sodium benzoate was found to be significantly superior to placebo in pain relief in patients with postdural puncture headache. Yücel et al. [39] reported that the administration of caffeine sodium benzoate intravenously significantly reduces postdural puncture headache in comparison to normal saline. However, these clinical trials were criticized due to their small sample size, methodological weaknesses and conflicting results, or invalid answers [40]. Ward et al. [41] observed that 65 mg and 130 mg of caffeine were superior to placebo in alleviating non-migrainous headaches. Myers et al. [42] observed a considerable analgesic efficacy of oral caffeine (200 mg) in human experimental ischemic muscle pain.

The effectiveness of caffeine (100 mg) as an analgesic adjuvant to ibuprofen (100 mg or 200 mg) was demonstrated by evaluating the ibuprofen-caffeine combination in the treatment of postoperative pain after removal of third molars [43]. It was found to increase the potency of ibuprofen by 140-180%. Caffeine (50 mg, 100 mg, 200 mg) increased the analgesic effect of ibuprofen 200 mg. There was also an earlier onset of analgesic effect, as was shown in another study (third molar extraction) [44]. There were no significant differences between the three caffeine treatments. A superior efficacy of the combination of ibuprofen (400 mg) and caffeine (200 mg) compared to ibuprofen (400 mg) alone, caffeine (200 mg) alone and placebo was shown in a study in patients with tension-type headache [45]. However, it should be noted that all these studies had methodological limitations, in that the primary endpoint was not defined a priori, and the significance level was not adjusted for multiple testing.

A double-blind cross-over pilot study evaluated the effect of ibuprofen (100-400 mg, dosage was selected depending on body weight) and caffeine (50-100 mg) compared with ibuprofen and placebo in 12 children with headaches. Although this small pilot study was not sufficiently powered to prove any statistically significant benefit for caffeine with ibuprofen, the study did show a trend toward a superior efficacy of the combination vs. ibuprofen as monotherapy [46] in children as well. The results of a clinical study suggest that the combination of diclofenac sodium (100 mg) and caffeine (100 mg) is also more effective than diclofenac sodium (100 mg) alone in the acute treatment of migraine [47].

Caffeine has been found to accentuate the analgesic effects of acetaminophen and ASA in a broad collection of pain states such as tension-type and migraine headaches, dysmenorrhoea, cancer pain, postpartum pain, sore throat, and dental postsurgery pain. For example, the combination of paracetamol (1000 mg) and caffeine (130 mg) was significantly more effective than paracetamol alone in the treatment of tension-type headache [48]. Paracetamol (1000 mg) combined with caffeine (130 mg) was found to be an effective treatment for primary dysmenorrhoea. Here caffeine acts as an analgesic adjuvant and enhances the efficacy of paracetamol [49]. In an experimental human pain model based on pain-related cortical potentials after phasic stimulation of nasal mucosa with CO_2 _and pain ratings after tonic stimulation with dry air, the combination of paracetamol (1000 mg) with caffeine (130 mg) was shown to be significantly superior compared to paracetamol (1000 mg) or caffeine (130 mg) alone in reducing perceived pain throughout the whole measurement period [50]. Paracetamol absorption was accelerated by caffeine, which confirms earlier findings [51]. In the same pain model, it had already been shown previously that caffeine (100-150 mg) enhances the analgesic activity of propyphenazone (400-600 mg) [52]. In the treatment of tension-type headache, the combination of paracetamol (1000 mg) with caffeine (130 mg) was significantly superior to placebo [53]. ASA (650 mg) in combination with caffeine (65 mg) was statistically superior to ASA alone in postoperative oral surgery pain [54], and ASA (800 mg) combined with caffeine (64 mg) was superior to ASA as a monotherapy in sore throat pain [55].

### 2.4 ASA + paracetamol + caffeine - A specific multi-target pain medication

#### 2.4.1 Clinical efficacy

A logical consequence of the arguments discussed above is that not only the combination of two substances was introduced but also the fixed combinations of caffeine with two analgesics were investigated. A meta-analysis of the effect of caffeine in combination with ASA and paracetamol involving more than 10,000 patients was conducted [56]. Although most studies included patients with postpartum uterine cramping or episiotomy pain, some comprised patients with pain from oral surgery or headache. The overall pooled relative potency estimate of 26 clinical dose-finding studies was 1.41; that is, to obtain the same amount of response from an analgesic without caffeine requires a dose that is approximately 40% greater than one with caffeine [56]. The optimal dose ratio of ASA: paracetamol: caffeine was found to be 1: 1: 0.25, with the minimum dose of caffeine being 50 mg [56].

One of the best examined fixed-dose combinations is the combination of ASA + paracetamol + caffeine for the treatment of headache. In six randomized, controlled double-blind studies (Table 2), this combination was superior both to placebo and to the control therapies sumatriptan (50 mg) [60], ibuprofen (400 mg) [61], ASA + paracetamol [57,59], ASA [57,59], paracetamol [59], and caffeine [59] in patients with migraine and/or tension-type headache in terms of their analgesic effectiveness. Criticism was levelled at Diener et al.'s [59] study that from a clinical point of view, the superiority of the combination is only modest. However, this disregards the fact that in this study, the comparison was not with the dosage of the individual active ingredients in the combination (here 500 mg ASA, 400 mg paracetamol), as is usual in studies designed to prove the combination rationale. Rather, the comparison was with the highest permitted dosage of 1000 mg ASA or 1000 mg paracetamol. Nevertheless, the combination still proved to be significantly superior in terms of its analgesic effectiveness. Moreover, irrespective of this, the clinical relevance of the observed differences in the efficacy endpoints in favour of the combination of ASA + paracetamol + caffeine was underpinned by the fact that these corresponded to the difference between "very good" and "good" in the global assessment of efficacy of patients [62].

**Table 2:** Clinical studies of the fixed combination of acetylsalicylic acid (ASA) + Paracetamol (PARA) and caffeine (CAF) (published 1988-2006)

Study	Study design	Indication	Results-efficacy endpoint
Bosse & Kühner 1988[57]	randomized double-blind, multicenter study	Headache of different genesis	ASA + PARA + CAF* > 250 mg ASA + 250 mg PARA ASA + PARA + CAF* > 500 mg ASA

Migliardi et al. 1994[48]	four identical, randomized double-blind, two-period crossover, multicenter studies	Tension-type headache	ASA + PARA + CAF** > 1000 mg PARA ASA + PARA + CAF** > Placebo

Lipton et al. 1998[58]	three randomized, double-blind, studies.	Migraine	ASA + PARA + CAF** > Placebo

Diener et al. 2005[59]	randomized double-blind, multicenter study	Migraine and tension-type headache	ASA + PARA + CAF*** > 500 mg ASA + 400 mg PARA ASA + PARA + CAF*** > 1000 mgASA ASA + PARA + CAF*** > 1000 mg PARA ASA + PARA + CAF*** > 100 mg CAF ASA + PARA + CAF*** > Placebo

Goldstein et al. 2005[60]	randomized double-blind, multicenter study	Migraine	ASA + PARA + CAF** > 50 mg Sumatriptan ASA + PARA + CAF** > Placebo

Goldstein et al. 2006[61]	randomized double-blind, multicenter study	Migraine	ASA + PARA + CAF** > 400 mg Ibuprofen ASA + PARA + CAF** > Placebo

*: 250 mg ASA + 200 mg PARA + 50 mg CAF; **: 500 mg ASA + 500 mg PARA + 130 mg CAF; ***: 500 mg ASA + 400 mg PARA + 100 mg CAF.

#### 2.4.2 Safety aspects of caffeine-containing analgesics

From the perspective of the drug regulatory authorities, "self-medication must have a low level of toxicity and low risk of serious adverse reactions. Proposed for the treatment of benign symptoms, these drugs must have a good benefit/risk ratio and the side effects have to be rare and not severe. Clinically significant interaction with other commonly used medicines or major prescribed drugs must be avoided. The nonprescription medicine must present no indirect danger, e.g. masking a condition requiring medical advice, and must not pose any risk of resistance for the population, e.g. antibiotics" [63]. Conversely, it holds that these requirements are fulfilled in preparations which have been approved by the regulatory authorities for self-medication, thus including the fixed combination ASA + paracetamol + caffeine. As early as 1993, caffeine in this combination was evaluated by the Non-Prescription Drug Advisory Board of the FDA as a category 1 analgesic adjuvant ("recognized as safe and effective") [64]. This evaluation corresponds to that of the German regulatory authority, the BGA (Bundesgesundheitsamt; German Health Authority), which was published in the form of two monographs in the "Bundesanzeiger" (the German Federal Gazette) [65,66].

##### 2.4.2.1 Analgesics and nephropathy

In spite of this, allegations have been repeatedly made in relation to caffeine-containing analgesics, with claims of an increased risk of nephropathy, and an induction or maintenance of increased or frequent use of these analgesics that does not comply with regulations. This is highly surprising, as three detailed analyses of the epidemiological studies [67-69] upon which the allegations of an increased risk of developing nephropathy were based showed, independently of one another, that these data do not conclusively establish a causal link between use of specific analgesics and chronic renal failure [68]. Bach et al. specify that the currently available animal and human data do not support the notion that the nephrotoxic risk from coformulated ASA or acetaminophen is higher than the risk from either ASA or acetaminophen alone, in equivalent analgesic doses. They add that there are no epidemiological data that implicate caffeine in analgesic-associated nephropathy [67]. In their analysis, Feinstein et al. [69] point in particular to the fact that in the studies available at the time, to which the bibliography of ICHD-2 (pp. 98-101) [70] refers, the problem of the influence of phenacetin, which had been a component of many combination analgesics for several decades, was only considered "very insufficiently", if at all. Thus, Feinstein et al. conclude that there is not sufficient evidence to associate nonphenacetin-combined analgesics with nephropathy, and that new studies should be conducted to provide appropriate data in order to resolve the question [69]. In the subsequently implemented autopsy study in Basel, based on over 600 consecutive autopsies, Mihatsch et al. [71] reached the conclusion that the classic analgesic nephropathy disappeared some 20 years after the removal of phenacetin from the analgesic market, despite the fact that mixed analgesics containing paracetamol, the main metabolite of phenacetin, have continued to be popular and widely used drugs [71]. The epidemiological case-control study conducted upon the suggestion of Feinstein et al. concluded that there was no clinically meaningful evidence for an increased risk of ESRD (end-stage renal disease) associated with the use of phenacetin-free analgesics in single or combined formulation [72]. Of the 907 cases, the 22 cases with the highest analgesic intake were evaluated individually, resulting in the conclusion that data supporting the existence of such an analgesic-associated nephropathy (AAN) are, however, not consistent and most likely due to confounding by indication [73]. These findings were supplemented by a prospective cohort study (Physicians' Health Study), which showed that no association was observed between analgesic use and reduced creatinine clearance (as a marker for renal dysfunction) [74].

##### 2.4.2.2 Caffeine and the overuse of analgesics

Regarding the issue of increased or frequent use, in the monographs of the German regulatory authority (BGA, now Bundesamt für Arzneimttel und Medizinprodukte BfArM; Federal Institute for Drugs and Medical Devices), it is clarified that there is no evidence that the potential for dependency on analgesics such as ASS (paracetamol) is increased by caffeine. Even if it can be assumed on the basis of theoretical considerations, an independent potential of abuse of caffeine in combination with ASS (paracetamol) is not substantiated by the scientific findings [65,66]. Accordingly, caffeine abuse or dependence was also not incorporated as a diagnosis in the DSM-IV and ICD-10 [75]. Thus, in answer to the question "does caffeine contribute to overuse or abuse of analgesic products?", Dalessio stated in his Editorial that "No, it does not!" [76].

Nevertheless, caffeine coformulated with analgesics has been repeatedly accused, particularly by nephrologists, of inducing or sustaining analgesic overuse. A detailed analysis of the original publications behind the numerous literature citations shows that the epidemiological studies do not provide convincing evidence for a role of caffeine in prompting excessive analgesic use. Moreover, the identified groups of nephrologists did not provide substantial data to advocate the said suspicion, except for the observation of a preferential choice of phenacetin-containing combinations, especially powder preparations [77]. The fact that phenacetin, which was used exclusively in the form of combination analgesics (often in conjunction with caffeine), is the sole antipyretic analgesic to be accorded with a drug seeking or craving behaviour, as described by Murray [78 and 79], has often been overlooked. Moreover, this effect was no longer observed once it had been replaced with paracetamol [77]. It was phenacetin rather than caffeine that induced overuse/abuse of combination analgesics and phenacetin, but paracetamol does not have this psychotropic potential, as Fox pointedly concluded [80]. Feinstein et al. [81] came to a very similar evaluation; using an experimental design, they established that caffeine does show a small dependence potential, but they emphasise the important point that experimental data regarding dependence potential for caffeine alone may not correspond to the conditions in patients with pain. Withdrawal is not likely to cause stimulation or sustainment of analgesic intake. Strong dependence behaviour was observed only in patients using phenacetin-containing preparations, coformulated with antipyretics/analgesics and caffeine. This finding may have led to the impression that caffeine stimulates overuse of analgesics [81].

##### 2.4.2.3 Medication overuse headache (MOH)

Although allegations of an increased MOH risk in caffeine combined headache medications did not find their way into ICHD-2, they are mentioned in the bibliography (pp. 98-101) of the ICHD-2 in Chapter 8.2, "Medication-overuse headache" [70]. On the other hand, Diener & Tfelt-Hansen stated in the 1st edition and Diener & Dahlöf in the 2nd edition of the textbook "The Headaches" [82,83], that prevalence and incidence rates of chronic drug-induced headache are not available. Therefore, the data that have been gathered are based mainly on clinical series describing patients presenting at headache clinics with this problem. As "the drugs leading to chronic drug-induced headache vary considerably in different series depending on both selection of patients and cultural factors", due to the selection bias, which cannot be assessed in terms of its effects, it is not possible to make generalising statements. Moreover, it is "difficult to identify a single substance as 90% of patients take more than one compound at a time" [82,83]. In the 3rd edition of the book, Diener and Silberstein [84] make it clear that, with regard to the chapter on MOH in "The Headaches", it should be pointed out that the data discussed do "not at all support any particular risk" of a fixed combination of ASA, paracetamol and caffeine for the development of MOH; therefore, "differentiating recommendations on this specific combination compared with the classical OTC single analgesics do not seem justified" [84].

As it has nevertheless been claimed from various perspectives, mostly on the basis of answering this question of unsuitable clinical case series, that caffeine-containing analgesics show a higher risk for the development of a drug-induced headache, Dalessio had already pointed out back in 1994 that "there is no evidence that analgesics with caffeine are more likely to produce or exacerbate headache than analgesics without caffeine" [76]. This was confirmed by the evaluation by Feinstein et al. that for "for drug-induced headache, no single or combined analgesic was consistently identified as causative, and no evidence exists for a special role of caffeine" [81].

However, in the literature, the prevalence of MOH is indicated as lying at ~ 1% of the general population [85,86]. The validity of these data is difficult to judge, because they do not represent MOH in accordance with ICHD-2, but instead, data on parameters such as headache frequency and frequency of use of migraine and headache medications were actually interpreted as an indication for MOH [87]. Even more seriously, none of the studies that are repeatedly given as references for MOH prevalence actually present MOH prevalence either literally or conceptually in the sense of MOH in accordance with ICHD-2 [87].

The recently published study by Scher et al. [88] compares medication use among people classified as having chronic daily headache with medication use among people with episodic headache. Results indicated weak positive associations (adjusted odds ratios) for past use for non-prescription caffeine-containing combination analgesics. Scher et al. interpreted their findings with caution because of methodological limitations, including lack of data on dose-response and possible recall bias, reverse causality and confounding by indication [89]. Furthermore, Delzell and Haag raise the concern that analyses were only adjusted for potential confounding by age, gender, primary headache type, recall year and number of medications. Because other risk factors for CDH have been identified (e.g. lower socioeconomic status, comorbid conditions causing pain, obesity, sleep disorders), residual confounding is possible if these uncontrolled factors were associated with use of specific medications [89]. Although Scher et al. did not explicitly discuss their results for nonprescription caffeine-containing combination analgesics, and nor did they conclude that such drugs are causally associated with CDH, Delzell and Haag stress that this was the only study that was drawn on in the new EFNS guideline on the treatment of tension-type headache in the evaluation of caffeine-containing combination analgesics [90]. This procedure is neither methodologically sound nor accurate in terms of content, as in other studies, such as the longitudinal study by Bigal et al. [91], which examined the question of whether different classes of medicines are associated with a differing risk of chronification of migraines, caffeine-containing analgesics did not show an increased risk. Moreover, this study refutes the claim, which had frequently been expressed in the past, that caffeine in combination analgesics induces a more frequent and/or longer intake of these preparations compared to monoanalgesics. Among the examined OTC analgesics (paracetamol, NSAIDS), the fixed combination of acetylsalicylic acid, paracetamol and caffeine was taken for the shortest period of time, both by persons with episodic migraine and by persons who developed a transformed migraine.

However, even more important is the fact that the ultimately decisive question, namely when an Odds Ratio (or relative risk) is to be evaluated as a "relevant risk", is clearly given too little attention compared to the clinical relevance of results of randomised, clinical effectiveness studies. In this regard, in a noteworthy publication in "Science" entitled "Epidemiology Faces Its Limits" [92] Taubes cites various experts who hold, among other things, that no epidemiological study taken by itself is convincing if the lower confidence limit does not show a relative risk of at least 3 or even 4. As a basic rule, it is recommended that the investigation be "forgotten" if the relative risk is not at least 3 or 4. The complex multivariate analytical procedure of epidemiological studies seems to foster purely quantifying, model-oriented "evaluations", and increasingly leaves out qualifying evaluations which take into account the limits of epidemiological methodology.

There is still considerable controversy still regarding the classification of individual headaches, including chronic migraine (CM) and medication overuse headache (MOH) [93]. It can be difficult to decide whether the regular overuse of pain medication is the cause or the result of the increased headache frequency [94]. Excessive medication use is a problem for some, but not all, migraine sufferers; therefore, medication use is a risk factor (increases the risk of a different disease state), and not a disease (which a person either has or does not have) [95]. Is medication-overuse headache a distinct biological entity? [96]. One can agree entirely with Bigal et al. when they state that the ultimate question that needs to be discussed is whether MOH should exist as a single entity or should be more appropriately viewed as a risk factor [95].

In a recently published cross-sectional study [97], which was one of the few studies to examine the association of a multitude of possible factors for the chronification of episodic migraine, only the consumption of hypnotics, pre-existing hypertension, a previous cranial trauma, parents with chronic migraine, sleep disorders, female gender, and an increased rating on the depression scale of Zung were found to be risk factors for a chronification. The factors did not include the "classical" risk factors such as headache frequency and frequent intake of migraine and headache medications.

### 2.5 Molecular targets of pain therapy - effects of a specific combined analgesic

As discussed in the introduction, multiple pathways and therefore molecular mechanisms are involved in the pathophysiology of pain (including headache) and are thus multiple potential targets for pharmacological pain therapy. Here, as an example of a precise multi-target OTC therapy, we summarize the effects of combined analgesics containing ASA, paracetamol and caffeine on major mediators of pain such as prostaglandins, the vanilloid and cannabinoid system and catecholamines.

#### 2.5.1. Prostaglandins

The central mode of action of non-steroidal analgesics (NSAID) including ASA is the inhibition of the cyclooxygenases (COX). ASA inhibits the two isoenzymes cyclooxygenase-1 (COX-1) and cyclooxygenase-2 (COX-2) dose-dependently, and consequently the synthesis of prostaglandins, although with higher selectivity for COX-1 than for COX-2.

The prostaglandin (PG) essential for pain and inflammation development is PGE2. It is generated during inflammation mainly by COX-2 and the prostaglandin E2 synthase mPGES-1. Besides its key role in pain and inflammation, PGE2 plays a central role in the elevation of body temperature (fever), which explains the antipyretic and antiphlogistic effect of COX inhibitors. The blockage of PGE2 synthesis by the COX inhibitors such as ASA and classical NSAIDs as well as the novel selective COX-2 inhibitors (coxibs) leads to substantial pain inhibition, which is strongest for inflammatory pain. PGE2 mediates its various effects by binding to four receptor subtypes - EP1 through EP4. Pain processes, depending on the tissue, seem to involve all EP receptors. By means of selective agonists/antagonists, a pain-inhibiting effect has been demonstrated for each receptor subtype depending on the tissue. EP2 and EP3 mediate the pain-inducing effect of PGE2 on peripheral nociceptors and in the medulla [98,99].

The binding of PGE2 to its receptors leads in many cases to an increase in cyclic AMPs (cAMPs) and in intracellular calcium, and to the activation of phospholipase C and proteinkinase A. As a consequence, excitatory ion channels can be opened in peripheral pain-sensitive nerve fibres, such as TRPV1 (transient receptor potential vanilloid 1). Furthermore, the inhibitory neurotransmission in the medulla mediated by glycin receptors can be blocked, which otherwise decrease nociceptive excitation [100,101]. There are suggestions that PGE2, via the EP2 receptor, can affect the synthesis of CGRP and thus also counteract pronociceptive actions of CGRP [102]. Modulation of CGRP-mediated effects at its receptor is the mechanism of a novel class of migraine medications known as nonpeptide competitive CGRP antagonists [103,104].

It is still not fully understood which mechanisms of paracetamol are responsible for its analgesic and antipyretic effect in humans [105]. In some cell types, inhibition of PGE2 synthesis can be shown [106], but the mechanisms cannot be completely explained, especially since paracetamol does not inhibit the synthesis of prostaglandins in many peripheral immuno-competent and inflammatory cells, because in these cells, high peroxide levels block cyclooxygenase inhibition by paracetamol [107,108]. In immunocompetent cells of the nervous system such as microglia, paracetamol, however, reduces PGE2 synthesis as effectively as ASA [107,109], whereas ASA increases this inhibition synergistically [109]. In contrast to peripheral immunocompetent cells, microglia show low peroxide levels that do not block the cyclooxygenase antagonism of paracetamol [105]. The fact that paracetamol enhances the effect of ASA and other NSAIDs [110] supports a differential mechanism of prostaglandin inhibition as compared with other NSAIDs, i.e. not a direct inhibition of the enzyme activity of the cyclooxygenases by paracetamol.

Caffeine has been shown to decrease prostaglandin synthesis in microglia cultures [109]. Unlike ASA or paracetamol, caffeine does not block the activity, but reduces de novo protein synthesis of the cyclooxygenases[109]. The fact that adenosine - via the A2a receptors and the formation of cyclic AMP - can prompt PGE2 synthesis in microglia cells suggests an additional mechanism for the analgesic effects of caffeine [111].

In a peripheral pain model, caffeine also enhances the analgesic and anti-inflammatory effect (hyperalgesia) of acetylsalicylic acid, but this effect is independent of COX/prostaglandin synthesis [112].

The combination of ASA, paracetamol and caffeine shows a synergistic inhibitory effect on PGE2 synthesis. How can this synergism be explained? While ASA inhibits the activity of cyclooxygenases directly and irreversibly, paracetamol decreases the activity of the cyclooxygenases in the nervous system, at least in the cells, such as microglia, with low peroxide levels [107]. In addition, caffeine mitigates the protein synthesis of COX-2. In this way, the triple combination enhances the inhibition of COX-2 activity.

#### 2.5.2 Cannabinoid and vanilloid receptors

The endogenous cannabinoid system is also part of the body's own protective function against headache. In females with migraine, the cannabinoid anandamide is metabolized increasingly [113], which physiologically alleviates nociception in the trigeminal nerve [114]. Cannabinoids also inhibit the peripheral vanilloid (TRPV1) receptors on neurons of the trigeminal nerve [115]. CB1 receptors, which lessen neuronal excitement and mediate the effect of paracetamol in animal models [116], can be activated by cyclooxygenase-2 inhibitors such as meloxicam [117].

In the nervous system, paracetamol is metabolized to AM404 [N-(4-hydroxyphenyl)-arachidonyl amide] by means of FAAH (fatty acid amide hydrolase) [118]. AM404 inhibits the anandamide membrane transporter (AMT) and thus endocannabinoid re-absorption, thereby elevating the endocannabinoid levels. Animal experiments show that the cannabinoid-1 receptor (CB1) mediates pain inhibition by paracetamol and its metabolite AN404, as the blockage of CB1 leads to a complete loss of the analgesic effect of paracetamol [119,120]. AMA404 also stimulates the TRPV1 receptor directly [105]. Contrary to the nociceptive TRPV1 receptors of the peripheral nerve endings (see above), the activation of central TRPV1 receptors after intrathecal administration of the endocannabinoid anandamide causes a significant suppression of the pain response in the medulla [118]. At higher doses requiring more than 1000 mg paracetamol, AM404 in vitro also inhibits COX-1 and COX-2 and decreases prostaglandin synthesis in macrophages [105].

CB-1 receptors can be activated by cyclooxygenase-2 inhibitors [117]. The combination of ASA + paracetamol + caffeine might be considered a functional axis between the stimulation of CB receptors and COX inhibition.

In summary, paracetamol and its metabolite AM404 possess some pleiotropic analgesic potential that also includes - in addition to COX-2 blockade - the modulation of serotonergic transmission and activation of the endocannabinoid system. The cannabinoid/vanilloid system does not seem to be affected by ASA and caffeine

#### 2.5.3 Catecholamines

Dopamine, noradrenaline, and adrenaline are involved in the control of nociception and analgesia in spinal and supraspinal nuclei [121,122]. The enhancement of noradrenergic transmission is the pathway by which tricyclic antidepressants, SNRI (serotonin-noradrenaline reuptake inhibitors), alpha2-adrenergic inhibitors, and alpha2-adrenergic mimetics take their clinically relevant analgesic effect [100,123]. Noradrenaline mimetics are among the most powerful co-analgesics for neuropathic pain [124]. In addition, noradrenaline inhibits inflammatory effects of microglia, which also lowers the pain process [125].

Modulation of serotonergic neurotransmission might be one mechanism of the analgesic effects of paracetamol. In animal experiments, paracetamol increased the serotonin level in the CNS [126] and (indirectly) activated 5-HT1A receptors [127,128] as well as 5-HT3 receptors [129]. This was verified in humans: the analgesic effect of paracetamol on an experimental pain stimulus was completely blocked in healthy subjects by IV application of the 5-HT3 antagonists tropistron or granisetron [130]. This is postulated as an indirect effect, since paracetamol does not bind to serotonin receptors.

Similar to paracetamol, there are also indications of a modulation of serotonergic transmission for caffeine. In an animal experiment, caffeine enhanced serotonin secretion [131] and the analgesic effect of clomipramine, a tricyclic antidepressant, with particularly pronounced inhibition of serotonin re-uptake [132], causing the synaptic availability of serotonin to rise.

However, caffeine also shows a central analgesic effect in the cholinergic system, probably by enhancing central cholinergic transmission [133].

xxxxx ab hier neue Ref Nr!!!

The combination of ASA + paracetamol + caffeine enhances noradrenaline synthesis in striatal brain sections of the rat, but reduces dopamine synthesis [134]. The differential secretion of catecholamines - more noradrenaline and less dopamine - from striatal brain sections suggests a novel mode of analgesic action of this combination. Derived from ex vivo findings in microglia, the combination is able to enhance the secretion of noradrenaline and thus induces analgesic efficacy. The inhibition of dopamine secretion is important because the activation of the dopamine system is considered an essential factor for cerebral blood flow and the post-dromal symptoms of migraine [135]. Several clinical trials describe a hypersensitization of peripheral and central-nervous dopamine receptors as a specific trait of migraine, as well as the activation of the dopamine system as the primary pathophysiological factor in certain migraine types [135].

By enhancing serotonergic transmission, paracetamol and caffeine can cause analgesia synergistically. By enhancing serotonergic transmission, paracetamol and caffeine can cause analgesia synergistically. An increase in serotonergic outflow under caffeine was also observed in a case history although this effect might be due to an interaction of caffeine and anti-depressants [136]. Additionally, there are indications of inhibition of the cyclooxygenases by the serotonergic system as a mechanism of analgesia [108].

### 2.6. Rationale for combined analgesics in (headache) pain therapy

The evidence from the clinical studies described above makes it clear that, in pain therapy, including therapy of primary headaches, hardly any single medicinal approach gives the impression that it could not be improved further in terms of its efficacy, safety, and/or tolerability by being combined with other substances. This applies to combinations of acidic and non-acidic antipyretic analgesics with each other, or with mild opioids or agonist-antagonist narcotics, as well as to their combination with antiemetics and/or caffeine.

As with the example of a fixed-dose combination of ASA, paracetamol (acetaminophen) and caffeine reviewed above, the major advantage of using such a combination is that the active ingredients act on different but distinct molecular targets and thus are able to act on more signalling cascades involved in pain than most single analgesics. Moreover, this leads to synergistic effects of the combination on pain mediators such as PGE_2_. This might explain their superior clinical efficacy compared to single analgesics and supports the hypothesis of a precise approach of combined analgesics resulting in a precise (molecular) mode of action.

Migraine therapy in particular provides an example of this approach. When triptans were introduced, monotherapy with these drugs supplanted the use of earlier combination pills of ergot, caffeine, tranquillizers or antiemetics in many countries [137]. However, combination therapy has played an increasingly important role in pain therapy, as, for example, in the WHO "analgesic ladder" concept for the management of cancer pain [138]. In their work "New drugs for migraine", it is obvious for Stovner and coauthors that the complex migraine pathophysiology offers multiple targets for pharmacological intervention, and today it is argued that drug combinations could provide additional effects compared to monotherapy [137]. The low rate of utilisation of triptans observed, which was associated with a high percentage of discontinuation, probably contributed to this evaluation [21,22]. Thus, Stovner et al. pick up on the arguments formulated by Peroutka in his work "Beyond monotherapy: rational polytherapy in migraine", that monotherapeutic approaches to migraine do not provide rapid consistent and complete relief in all migraineurs [20]. Therefore, if monotherapy is suboptimal, it logically follows that concurrent therapy (i.e., polytherapy) aimed at modulating two or three of the biological systems should be more efficacious than a therapy modulating only a single system [20]. His discussion ends with the conclusion that, fortunately, significant recent scientific progress has led to the ability to develop rational polytherapeutic regimens in migraine [20]. Stovner et al. add that we are also seeing a movement towards more polypharmacy with combinations of well-known drugs" [137].

The systematic investigation of multicomponent therapeutics is still in the early stages. This is based on the fact that a huge number of possible combinations exist, as illustrated by the following calculation: for a set of n compounds, binary combination space is described by n*(n-1)/2. For example, a set of 2,000 compounds has nearly 2 million possible binary combinations and many more higher-order combinations. Therefore, even a relatively small compound library, such as the set of approved drugs, yields a large number of combinations to be tested [8,139].

Recognition of the potential for multi-target intervention in biology and medicine has a long history [139]. The concepts of synergy, additivism, and antagonism have been explored extensively [139]. Non-interactive combinations are often said to show additivism. This usage is confusing because it is often taken to mean that the effects of such combinations may be obtained by adding the effects of their constituents, but this is true only in particular circumstances, [140]. Synergy broadly means "working together" and antagonism means "working against each other", but these generally understood meanings are not unambiguous definitions, and some quantitative criterion is clearly implied [141]. The crux of the matter is to decide what is expected, and various rules have been proposed to this end (for example, that the expected effect is the sum of the effects of the individual constituents of the combination, or that it is the product of these effects) [140]. These rules are valid for combinations of agents with particular and rather restricted types of dose-effect relations, but they have no general validity [140]. Non-interactive combinations are often said to show additivism. According to Berenbaum, this usage is confusing because it is often taken to mean that the effects of such combinations may be obtained by adding the effects of their constituents [140]. This assumption is valid only when all the agents in the combination show linear dose-effect relations, notes Berenbaum [140]. However, in pain therapy, the largest class of nonopioids, the NSAIDs, show a flat dose-response [44], a maximal dose or marked ceiling (plateau) effects [143]. Greater NSAID doses provide no additional analgesia [142]. For headache therapy, Diener et al. [59] also point out that an underestimated aspect of antipyretic analgesics, including NSAIDs, is the surprisingly flat dose-effect curve with a ceiling effect, which applies to both the acidic and non-acidic antipyretic analgesics [59]. The combination with caffeine, by contrast, increases the potency of ASA and paracetamol [59].

Keith et al. recently suggested the following terminology for combination drugs:

- Syncretic combination drug, to denote a drug that is composed of two or more active ingredients, at least one of which is not used individually to treat the target disease indication.

- A congruous combination drug is composed of two or more active ingredients, each of which has been individually used to treat the target disease indication.

- A multicomponent therapeutic is an optimized combination and formulation of multiple active ingredients. This category includes both syncretic and congruous drugs [8]. Multi-target therapeutics are among the most promising avenues toward treating multifactorial diseases [7]. Nevertheless, there are concerns that synergistic therapies would induce synergistic toxicity, as would be expected if the mechanisms that cause side effects are closely related to those involved with efficacy [7]. However, findings are accumulating that synergistic drug combinations are generally more specific to particular cellular contexts than are single agent activities [7,9]. Synergistic combinations of two or more agents can overcome toxicity and other side effects associated with high doses of single drugs by countering biological compensation, allowing reduced dosage of each compound, or accessing context-specific multi-target mechanisms [7].

The best definition of synergy for multicomponent therapeutics must reflect the physician's requirement that, when the drugs are used in combination, a benefit is observed that could not be achieved by the constituents on their own and that these act together and cooperate to achieve a completeness of the desired therapeutic effect [8]. As an example of this, the combination with caffeine is mentioned. Caffeine strengthens not only the analgesic effect of NSAIDs, ASA and paracetamol, but also eliminates possible sedative effects that can be evoked by various analgesics [143]. Together with its light mood-elevating effects, this contributes to the completeness of the desired therapeutic effect for patients suffering from pain, and is thus useful [144]. Above 200 to 300 mg of caffeine, dysphoria, motor unrest, nausea and vomiting occur [144], meaning that caffeine use should be subject to a self-limitation, and thus, as Forth states, it "makes little sense to allege that a person resorts more to painkillers due to additional caffeine" [145], particularly as caffeine is available in the form of OTC drugs in many countries. In this respect, the primary sources of caffeine, namely food and drink, including the widespread "energy drinks" have not yet even been taken into account. A more in-depth discussion of fixed-dose combinations can be found in the publication by Edwards, McQuay and Moore [146].

## 3. Summary

It can be established that several lines of evidence now suggest that the traditional notion of "one drug-one protein" for one disease no longer holds, and that treatment of most complex diseases can best be attempted using polypharmacological approaches [147]. It is now time to revisit past experiences to identify multicomponent therapeutics for the treatment of complex diseases [8]. The advances made in developing promiscuous drugs by following the paradigm of polypharmacology are becoming increasingly apparent, and the advantages of these drugs over traditional drugs in targeting diseases are being revealed [8,147]. This needs to be further proven in well-designed clinical studies. Particularly in headache therapy, multi-target combinations, such as triptan + NSAIDs, NSAIDs + caffeine, NSAID + phenothiazine + caffeine, and ASA + paracetamol + caffeine as prescription or OTC medication, provide a guarantee of a precise therapeutic multi-target approach. They broaden the array of therapeutic options, enable the completeness of the therapeutic effect, and allow doctors (and in self-medication with OTC medications the patients themselves) to customize treatment to the patient's specific needs [11]. Generally speaking, there is no evidence that multicomponent drugs generally bear a higher risk for adverse effects and on the other hand there is substantial clinical evidence that such a multicomponent therapy is even more effective than monocomponent therapies.

## List of abbreviations used

AAN: analgesic-associated nephropathy; ASA: acetylsalicylic acid; BGA: Bundesgesundheitsamt (former German regulatory authority), CDH: chronic daily headache; COX: cyclooxygenase; CNS: central nervous system, ESRD: end-stage renal disease; FAAH (fatty acid amide hydrolase); FDA: Federal Drug Agency (USA); HMO: Health Maintenance Organization; ICHD-2: International Classification of Headache Disorders 2^nd ^edition, MOH: medication overuse headache; NMDA: N-methyl-D-aspartate; NSAID: nonsteroidal anti-inflammatory drugs; OTC: over-the-counter; SNRI: serotonin-noradrenaline reuptake inhibitors; TTH: tension-type headache.

## Competing interests

AS received honoraria for participation in clinical trials, contribution to advisory boards or oral presentations from: Allergan Germany and Europe; Berlin Chemie, Germany; Boehringer Ingelheim, Germany; Desitin, Germany; MSD, Germany; Pfizer, Germany; and scientific grants from the German Research Council, the BMBF, and the Kröner-Fresenius-Foundation. BA is an employee of Boehringer Ingelheim Pharma GmbH & Co. KG, Germany. BF received honoraria for participation in research projects, clinical trials, contribution to advisory boards or oral presentations from: Böhringer Ingelheim, Germany; Novartis GmbH, Germany; Pascoe pharmazeutische Praeparate GmbH, Germany; and Steigerwald Arzneimittelwerk GmbH, Germany. GH received honoraria for participation in clinical trials, contribution to advisory boards or oral presentations from: Berlin-Chemie, Germany; Boehringer Ingelheim, Germany; Glaxo Smith Kline, Germany; Janssen Cilag, Germany; and MSD, Germany.

## Authors' contributions

AS was involved in drafting the manuscript, interpretation of the literature and critical revision. BA was involved in drafting the manuscript, literature searches, interpretation of the literature and critical revision. BF was involved in drafting the manuscript, literature searches, interpretation of the literature and critical revision. GH was involved in interpretation of the literature, literature searches, and critical revision. All authors read and approved the final manuscript.

## Pre-publication history

The pre-publication history for this paper can be accessed here:

http://www.biomedcentral.com/1471-2377/11/43/prepub
